# PilVax – a novel peptide delivery platform for the development of mucosal vaccines

**DOI:** 10.1038/s41598-018-20863-7

**Published:** 2018-02-07

**Authors:** Dasun Wagachchi, Jia-Yun C. Tsai, Callum Chalmers, Sam Blanchett, Jacelyn M. S. Loh, Thomas Proft

**Affiliations:** 1 Department of Molecular Medicine & Pathology, School of Medical Sciences, The University of Auckland, Auckland, 1023 New Zealand; 2 Maurice Wilkins Centre for Molecular Biodiscovery, Auckland, 1023 New Zealand

## Abstract

Peptide vaccines are an attractive strategy to engineer the induction of highly targeted immune responses and avoid potentially allergenic and/or reactogenic protein regions. However, peptides by themselves are often unstable and poorly immunogenic, necessitating the need for an adjuvant and a specialised delivery system. We have developed a novel peptide delivery platform (PilVax) that allows the presentation of a stabilised and highly amplified peptide as part of the group A streptococcus serotype M1 pilus structure (PilM1) on the surface of the non-pathogenic bacterium *Lactococcus lactis*. To show proof of concept, we have successfully inserted the model peptide Ova_324–339_ into 3 different loop regions of the backbone protein Spy0128, which resulted in the assembly of the pilus containing large numbers of peptide on the surface of *L*. *lactis*. Intranasal immunisation of mice with *L*. *lactis* PilM1-Ova generated measurable Ova-specific systemic and mucosal responses (IgA and IgG). Furthermore, we show that multiple peptides can be inserted into the PilVax platform and that peptides can also be incorporated into structurally similar, but antigenically different pilus structures. PilVax may be useful as a cost-effective platform for the development of peptide vaccines against a variety of important human pathogens.

## Introduction

Vaccines remain the most cost-effective and feasible means of infectious disease control in the community. They have resulted in the eradication of small pox and polio, and a substantial reduction in the incidence of other serious diseases such as measles, tetanus and diphtheria^1^. Vaccine development has progressed from live-attenuated or inactivated forms of microbial pathogens to subunit vaccines using one or a few selected proteins to elicit a protective immune response^2,3^. These formulations are generally regarded as the safer options, as many proteins within a whole organism are not only unnecessary for protective immunity, but often also carry the risk of triggering allergic or autoreactive responses^4^. More recently, peptide vaccines have been introduced as an alternative strategy. Short peptides can be synthesised that carry only the necessary epitopes for highly specific T cell and/or B cell responses. Unfortunately, peptides by themselves are often not very immunogenic and prone to proteolytic digestion^5,6^. Consequently, peptide vaccines require time-consuming and expensive chemical coupling to carriers for delivery and adjuvancy^4,7,8^.

Here we present a novel peptide delivery platform (PilVax) that uses a *Streptococcus pyogenes* (Group A Streptococcus, GAS) pilus expressed on the surface of *Lactococcus lactis*. Pili (*sing*. pilus) are hair-like peritrichous protrusions from the cell surface of a variety of Gram-positive and Gram-negative bacteria, with a main, but not exclusive function in host tissue adhesion^9,10^. In Gram-positive bacteria, such as *S*. *pyogenes*, pili usually consist of 3 structural proteins that are covalently linked to each other by a specialised pilus-assembly sortase. During assembly, a single tip adhesin is linked to a backbone pilin monomer, followed by integration of >100 additional backbone monomers to form the elongated fibre. Pilus extension is terminated after insertion of an adapter protein that is then recognised by the house-keeping sortase A and bound to the cell wall peptidoglycan^11^.

The pilus encoded in the **F**ibronectin- and **C**ollagen-binding **T** antigen-2 (FCT-2) region of serotype M1 strains^12^ is thus far the best-characterised GAS pilus. Collagen-binding protein (Cpa, Spy0125) is the tip adhesin that confers the adhesive properties of the pilus structure^13,14^. Spy0128 (FctA, T-antigen) is the backbone pilin that generates the pilus fibre^15^, and Spy0130 (FctB) is the anchor protein that links the FCT-2 pilus to the cell wall^16^. The crystal structure of the FCT-2 type pilus backbone protein (Spy0128) revealed an immunoglobulin-like fold with intramolecular isopeptide bonds resulting in a protease-resistant and very stable protein conformation^15^. Linkage between Spy0128 monomers is achieved by a transpeptidase (sortase C, Spy0129) reaction between the carboxyl group of a C-terminal threonine and the ε-amino group of an internal lysine, leaving the N-terminus protruding along the pilus fibre^15^. The pili are distributed over the entire GAS cell surface^17^.

Mucosal routes for vaccine delivery have gained increased interest in modern vaccinology. More recently, non-pathogenic food grade bacteria, such as *L*. *lactis* were shown to work as safe and efficacious live antigen carriers that elicit specific and protective immune responses against the target protein^18–22^. *L*. *lactis* secreting human IL-10 has also been used in a phase I clinical trial to treat patients with Crohn’s disease^23^.

It was shown previously that complete *S*. *pyogenes* pilus structures can be expressed on the surface of *L*. *lactis*^24–28^. Furthermore, it was reported that a model protein, maltose-binding protein (MBP), fused to the tip pilin of a GAS serotype M3 strain was able to induce mucosal MBP-specific antibody responses, showing proof-of-concept for a potential vaccine strategy^27^.

The aim of our study was to demonstrate that *L*. *lactis* bacteria expressing a complete GAS pilus structure can be used as a platform to carry stabilised peptide antigens for the generation of mucosal vaccines against selected human pathogens. We show that the model peptide Ova_324–339_, a well characterised B cell epitope^29,30^ inserted at suitable regions of the protease-resistant backbone pilus protein can be stably expressed in large numbers as part of the multimeric GAS pilus structure on the surface of *L*. *lactis* (Supplementary Figure 1) and triggers peptide-specific mucosal immune responses after intranasal immunisation of mice.

## Results

### Insertion of a model peptide into selected regions within Spy0128

**Figure 1:**
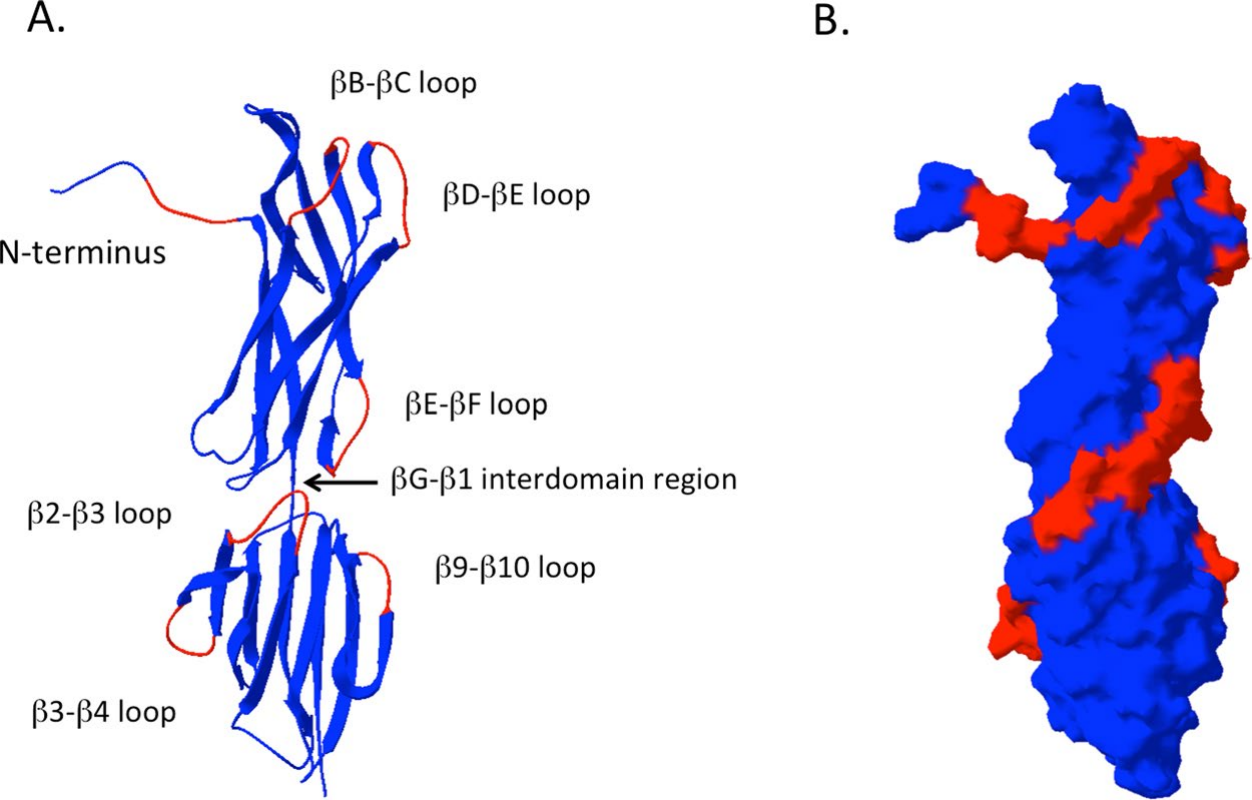
Protein structure of the backbone pilin Spy0128 with selected peptide insertion sites. The protein structure was generated with the PDB Swiss Viewer version 4.1.0.^54^ using coordinates downloaded from the Brookhaven database (3B2M). The selected peptide insertion regions are shown in red. (**A**) Ribbon diagram structure. (**B**) Accessible surface structure.

The GAS serotype M1 pilus (PilM1) encoded in the FCT-2 region has been extensively characterised^13,14,16,17^ and the crystal structure of the pilus backbone protein Spy0128 has been solved^15^. In order to identify potential integration sites for peptide epitopes, the protein structure of Spy0128 was analysed for surface accessible non-structured loop regions (Fig. 1). Based on this analysis, we selected 6 different loop regions for peptide integration. These include the βB-βC loop, the βD-βE loop and the βE-βF loop in the N-terminal domain and the β2-β3 loop, the β3-β4 loop and the β9-β10 loop in the C-terminal domain (Fig. 1). Furthermore, the free Spy0128 N-terminus and the region between the two Spy0128 domains (βG-β1 interdomain region) were also selected (Fig. 1). As the interdomain region is not well exposed we also inserted a peptide linker at this position.

**Figure 2:**
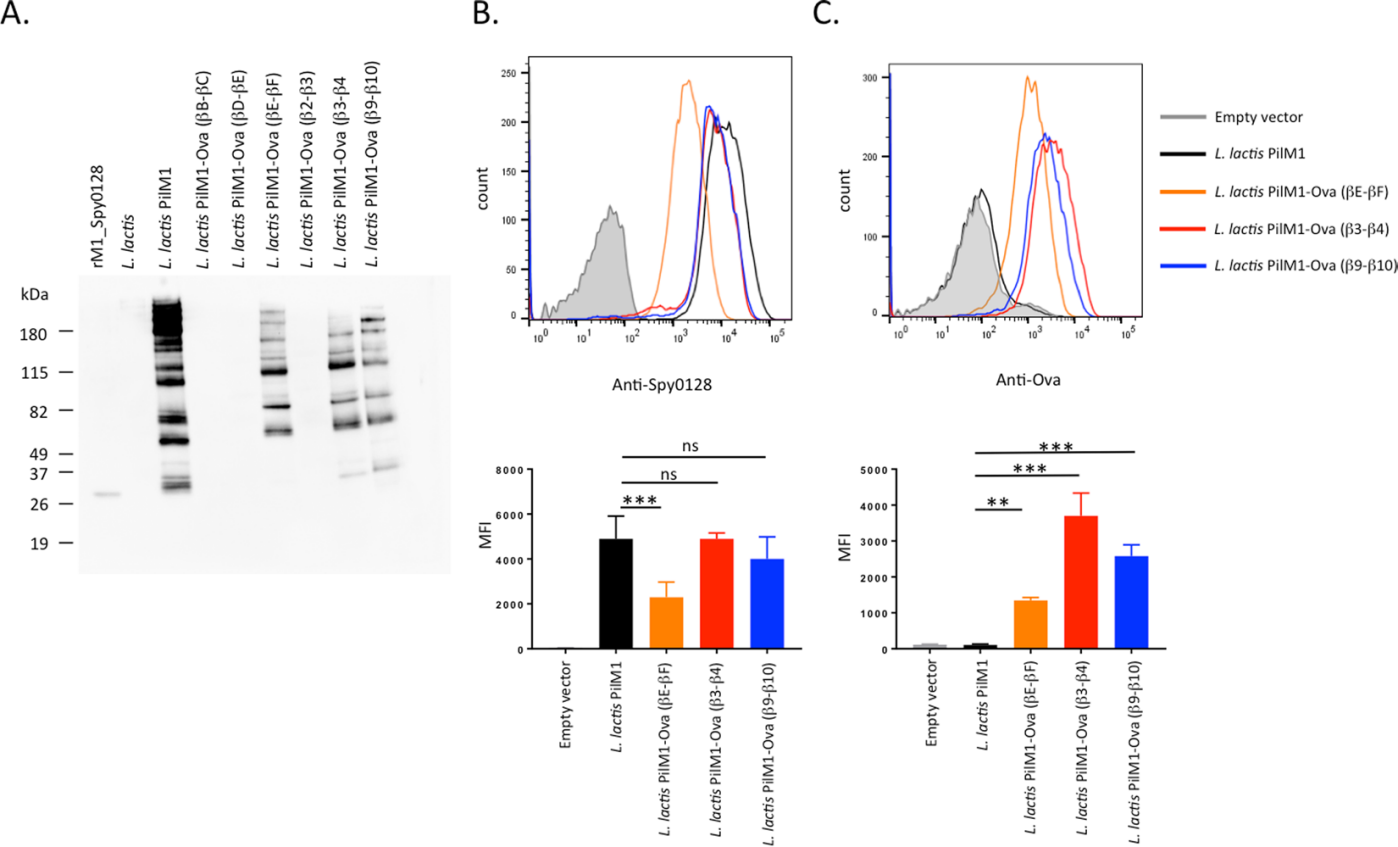
Expression of the PilM1 structure on the surface of *L*. *lactis* with the model peptide Ova_324–339_ inserted at selected sites within the Spy0128 backbone pilus protein. (**A**) Western blot analysis of *L*. *lactis* cell wall extracts (CWE) with antiserum specific for M1_Spy0128 (pilus backbone protein). The high molecular band patterns are indicative of pilus assembly. For *L*. *lactis* strains that showed pilus expression after peptide insertion, flow cytometry was used to compare the expression levels of M1_Spy0128 (**B**) and Ova_324–339_ peptide (**C**). Error bars show the standard deviation from 3 independent experiments. **p<0.005; ***p<0.0005; one-way ANOVA followed by a Holm-Sidak test.

As Spy0128 is expressed as a precursor with a N-terminal signal peptidase domain and a predicted cleavage site between Gly23 and Glu24, the N-terminus peptide integration site was introduced between Asn28 and Gly29. Table 1 shows the amino acid sequences of all the targeted regions before and after the introduction of the model peptide. The complete pilus structure was expressed in the food-grade bacterium *L*. *lactis* under the control of the constitutive P23 lactococcal promotor. To evaluate the successful assembly of the modified pili, *L*. *lactis* cell wall extracts were analysed in Western blots using specific anti-M1_Spy0128 antibodies (Fig. 2). Correctly assembled pili appear as a high molecular weight (HMW) laddering pattern due to the covalent linkage of variable numbers of individual pilin monomers, as seen for *L*. *lactis* expressing the unmodified M1 pilus (Fig. 2A). Unfortunately, insertion of the Ova_324–339_ peptide into the N-terminal region or the βG-β1interdomain region of Spy0128 did not result in expression of a pilus structure (Supplementary Figure 2). The addition of a GSGSG linker region on both ends of the Ova_324–339_ peptide in the βG-β1interdomain region also failed to produce pili (data not shown). Introduction of the Ova_324–339_ peptide into the βB-βC, the βD-βE, and the β2-β3 loops also affected pilus assembly (Fig. 2A). Western-blot analysis of the individual cell fractions revealed that *L*. *lactis* with Ova-peptide engineered into the βC-βD, β2-β3 loops or the interdomain region (βG-β1) expressed monomeric Spy0128 protein in the protoplast fraction with a slightly higher molecular weight than rSpy0128. In contrast, *L*. *lactis* with Ova-peptide at the βD-βE or the N-terminal region did not show Spy0128 protein in any cell fraction (Supplementary Figure 2). However, insertion of the Ova_324–339_ peptide into the βE-βF, the β3-β4 or β9-β10 loop regions resulted in assembled pili, indicated by a high-molecular weight banding pattern on Western blots with anti-M1_Spy0128 antibodies (Fig. 2A). Flow cytometry was used to investigate whether or not peptide integration in the βE-βF loop, the β3-β4 loop or the β9-β10 loop had an effect on the amount of Spy0128 pilus proteins expressed on the cell surface of *L*. *lactis*. As shown in Fig. 2B, the choice of peptide insertion site had a notable effect on pilus expression. Insertion of Ova_324–339_ peptide into the β3-β4 loop or the β9-β10 loop only slightly reduced Spy0128 expression, whereas the *L*. *lactis* strain with Ova_324–339_ peptide at the βE-βF loop expressed considerably less Spy0128 compared to wild-type. This was further confirmed by flow cytometry analysis using antibodies against the Ova_324–339_ peptide (Fig. 2C).

**Table 1:** Amino acid sequences of the peptide insertion sites in Spy0128.

Site	Original sequence	Sequence after peptide insertion
**a) M1_Spy0128**		
N-terminus	…TVVNGAK…	…TVVN**LESQAVHAAHAEINEAGRVE**GAK…
βG- β1_Ova	…SLDSTTLT…	…SLD**LESQAVHAAHAEINEAGRVE**TTL…
βG- β1-L	…SLDSTTLT…	…SLD**LD**GSGSG**LE**GSGSG**VE**TTL…
βG- β1-L_Ova	…SLDSTTLT…	…SLD**LD**GSGSG**LESQVHAAHAEINEAGRVE**GSGSG**VE**TTL…
βB-βC_Ova	…PDTTVNEDGNK…	…PDT**LESQAVHAAHAEINEAGRVE**GNK…
βD-βE_Ova	…FDFSEVTFEKPGVYY…	…FDF**LESQAVHAAHAEINEAGRVE**GVYY…
βE-βF_Ova	…TEEKIDKVPGVS…	…TEE**LESQAVHAAHAEINEAGRVE**GVS…
β2-β3_Ova	…LKANQYYKASEK…	…LKA**LESQAVHAAHAEINEAGRVE**SEK…
β3-β4_Ova	…KTTKGGQAPVQ…	…KTT**LESQAVHAAHAEINEAGRVE**PVQ…
β9-β10_Ova	…SPQDGAVKNI…	…SPQ**LESQAVHAAHAEINEAGRVE**KNI…
βE-βF_ ova-J14	…TEEKIDKVPGVS…	…TEE**LESQAVHAAHAEINEAGRVEKQAEDKVKASREAKKQVEKALEQLEDKVQVE**GVS…
**b) M18_Spy0128**		
βE-βF_Ova	…SEVNGNKAGIAY…	…SEV**LESQAVHAAHAEINEAGRVE**IAY…

The Ova_324–339_ and J14 peptide sequences are shown in bold underlined. Amino acids deriving from restriction enzyme recognition sites are shown in bold. Note that cloning of a *SalI* site (GTCGAC) into a *XhoI* site (CTCGAG) results in the hybrid sequence GTCGAG encoding amino acids VE. Peptide linker regions are underlined.

### Intranasal immunisation generates mucosal and systemic antibody responses in mice

**Figure 3:**
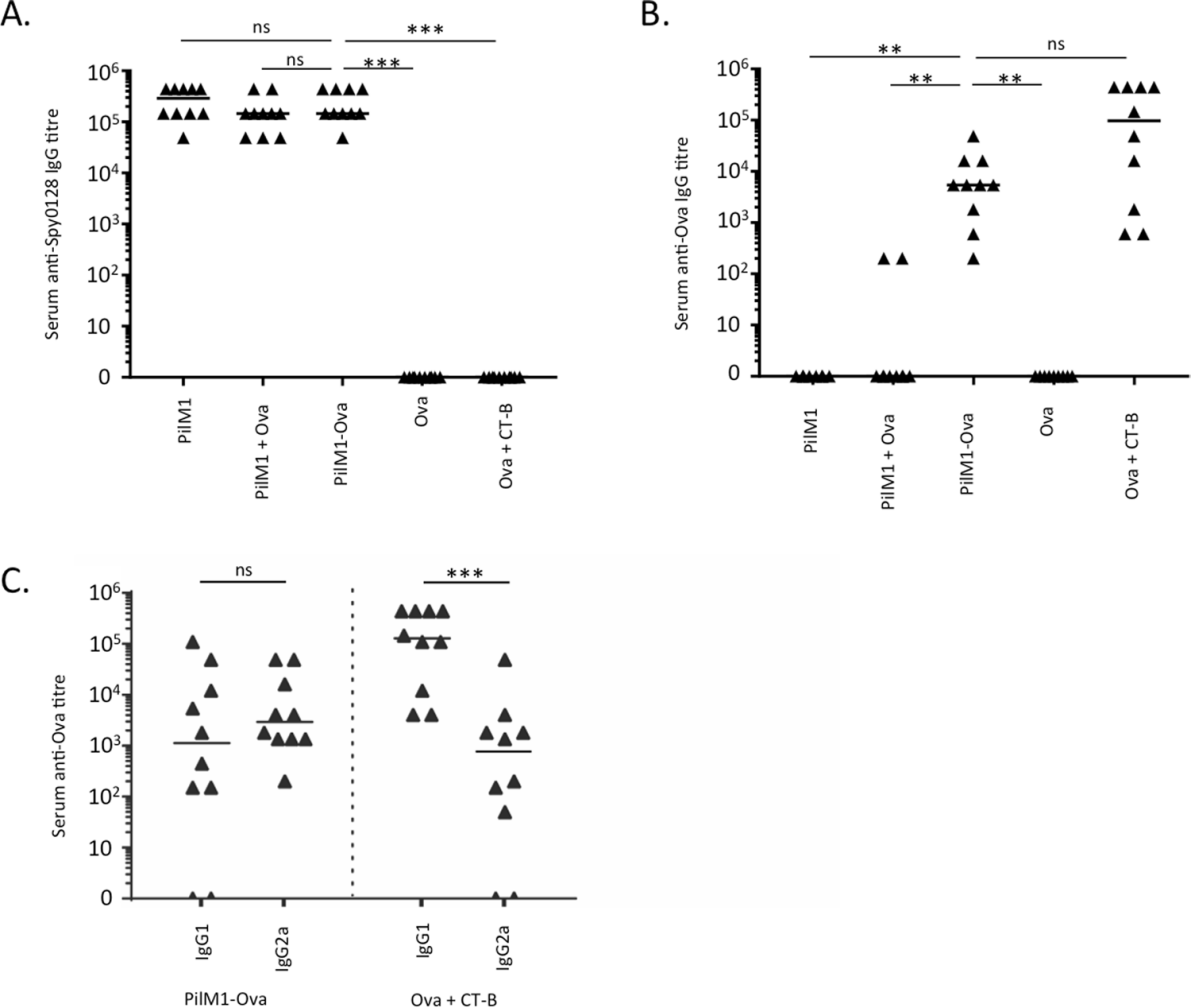
Intranasal immunisation of mice with *L*. *lactis* that expresses the M1 pilus with the model peptide Ova_324–339_ inserted at the βE-βF loop region (PilM1-Ova) induces IgG responses. Groups of Balb/c mice (n = 5) were immunised intranasally with 1 × 10^8^ CFU live recombinant *L*. *lactis* PilM1-Ova. The data from two independent experiments were combined. *L*. *lactis* without inserted peptide (PilM1) or mixed with synthetic Ova_324–339_ (PilM1 + Ova) were used as controls. Synthetic Ova_324–339_ peptide alone (Ova) or mixed with Cholera Toxin B subunit adjuvant (Ova + CT-B) were used as additional controls. Total serum IgG titres against (**A**) immobilised recombinant Spy0128 or (**B**) commercial ovalbumin were measured by ELISA. **p < 0.005; ***p < 0.0005; ns = not significant; One-way ANOVA with Dunn’s multiple comparisons test. (**C**) Ova-specific responses by IgG subclasses. ***p < 0.0005; Mann-Whitney test.

After successful integration of the Ova_324–339_ peptide into the Spy0128 pilus backbone protein and confirmed assembly of the modified pilus structure on the surface of the food-grade bacterium *L*. *lactis*, we were able to test our hypothesis that this peptide delivery platform could be used as a strategy to generate mucosal immune responses. Mice were immunised intranasally with live *L*. *lactis* bacteria that expressed the M1 pilus with Ova_324–339_ peptide inserted into the βE-βF loop region of Spy0128 (*L*. *lactis* PilM1-Ova). *L*. *lactis* expressing the unmodified M1 pilus without the peptide (*L*. *lactis* PilM1), *L*. *lactis* PilM1 mixed with synthetic Ova_324–339_ peptide (*L*. *lactis* PilM1 + Ova) and synthetic Ova_324–339_ peptide (with or without CT-B as an adjuvant) were used as additional controls. After 3 additional booster immunisations at weeks 2, 4, and 6, serum, saliva and bronchoalveolar lavage (BAL) fluid samples were obtained and analysed for Spy0128 and Ova-specific antibodies. Mice immunised with *L*. *lactis* PilM1, *L*. *lactis* PilM1 + Ova or *L*. *lactis* PilM1-Ova showed evenly high Spy0128-specific IgG titres (5 × 10^4^ to 5 × 10^6^) in serum samples, confirming reliable and consistent administration of the bacterial suspensions (Fig. 3A). Immunisation with *L*. *lactis* PilM1-Ova or synthetic Ova_324–339_ administered with the mucosal adjuvant CT-B, resulted in measurable and significant Ova-specific serum IgG responses (titres of 5 × 10^3^ to 1 × 10^4^) compared to mice immunised with the *L*. *lactis* PilM1 or *L*. *lactis* PilM1 + Ova controls (p < 0.005), which did not generate significant anti-Ova responses (Fig. 3B). Administration of synthetic Ova_324–339_ without CT-B also failed to generate Ova-specific IgG (p < 0.005). Notably, strong IgG responses were also seen with synthetic Ova_324–339_ when administered with CT-B. However, it should be mentioned that the amount of synthetic Ova_324–339_ was approximately 36,000 times higher than the amount of Ova_324–339_ peptide displayed on the surface of *L*. *lactis* PilM1-Ova.

To investigate if *L*. *lactis* PilM1-Ova preferentially targets specific IgG subclasses, we tested IgG1 and IgG2a responses in serum from vaccinated mice (Fig. 3C). There was no significant difference between IgG1 and IgG2a responses when mice were vaccinated with *L*. *lactis* PilM1-Ova. However, mice vaccinated with synthetic Ova_324–339_ plus CT-B generated significantly more IgG1 than IgG2a (p < 0.05).

**Figure 4:**
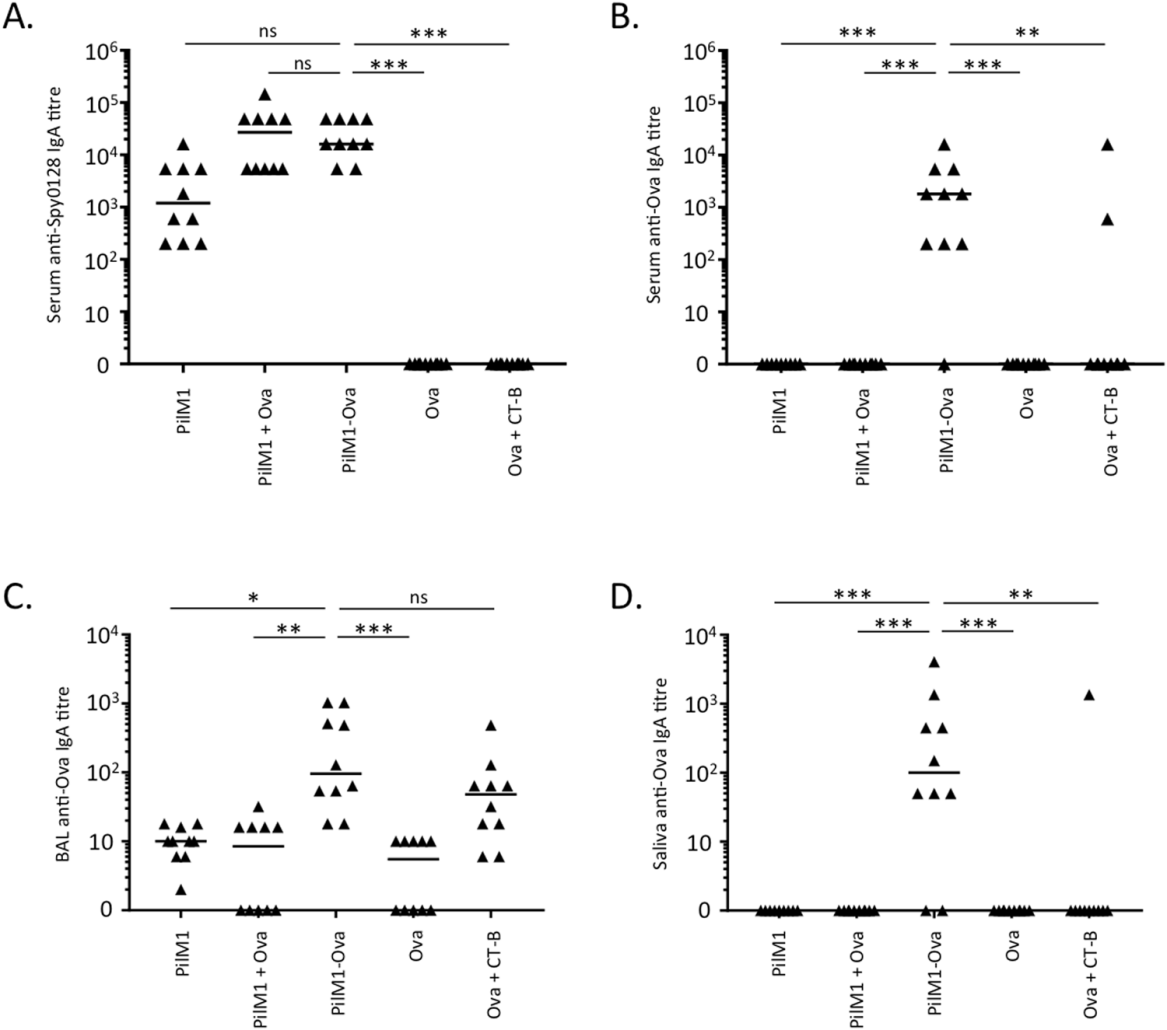
Intranasal immunisation of mice with *L*. *lactis* PilM1-Ova induces systemic and mucosal IgA responses. Groups of Balb/c mice (n = 5) were immunised intranasally with 1 × 10^8^ CFU live recombinant *L*. *lactis* PilM1-Ova. The data from two independent experiments were combined. *L*. *lactis* without inserted peptide (PilM1) or mixed with synthetic Ova_324–339_ (PilM1 + Ova) were used as controls. Synthetic Ova_324–339_ peptide alone (Ova) or mixed with Cholera Toxin B subunit adjuvant (Ova + CT-B) were used as additional controls. (**A**) Specific IgA responses against recombinant Spy0128 were measured by ELISA. ELISAs were also performed to measure specific anti-ovalbumin IgA responses in serum (**B**), BAL fluid (**C**) and saliva (**D**). *p < 0.05; **p < 0.005; ***p < 0.0005; ns = not significant; One-way ANOVA with Dunn’s multiple comparisons test.

As intranasal immunisation is expected to also generate mucosal responses, IgA titres were measured in serum (Fig. 4A and B), BAL fluid (Fig. 4C) and saliva (Fig. 4D). As seen for IgG, consistent IgA responses in serum of *L*. *lactis* PilM1, *L*. *lactis* PilM1 + Ova and *L*. *lactis* PilM1-Ova groups were detected against Spy0128, with titres of approximately 10^3^ to 5 × 10^4^ (Fig. 4A). Importantly, significant Ova-specific serum IgA responses were found in most mice immunised with *L*. *lactis* PilM1-Ova (titres ≥ 10^3^, p < 0.0005). In contrast, mice immunised with *L*. *lactis* PilM1, *L*. *lactis* PilM1 + Ova or synthetic Ova_324–339_ peptide (even when mixed with CT-B) all failed to generate detectable Ova-specific serum IgA (Fig. 4B). Similar responses were also seen in BAL fluid and saliva samples, although IgA titres were generally lower (Fig. 4C and D). The only difference was that Ova_324–339_ peptide plus CT-B induced Ova-specific IgA responses in BAL fluid, at a similar level to mice immunised with *L*. *lactis* PilM1-Ova.

### Expression of multiple peptides in the PilM1 pilus

**Figure 5:**
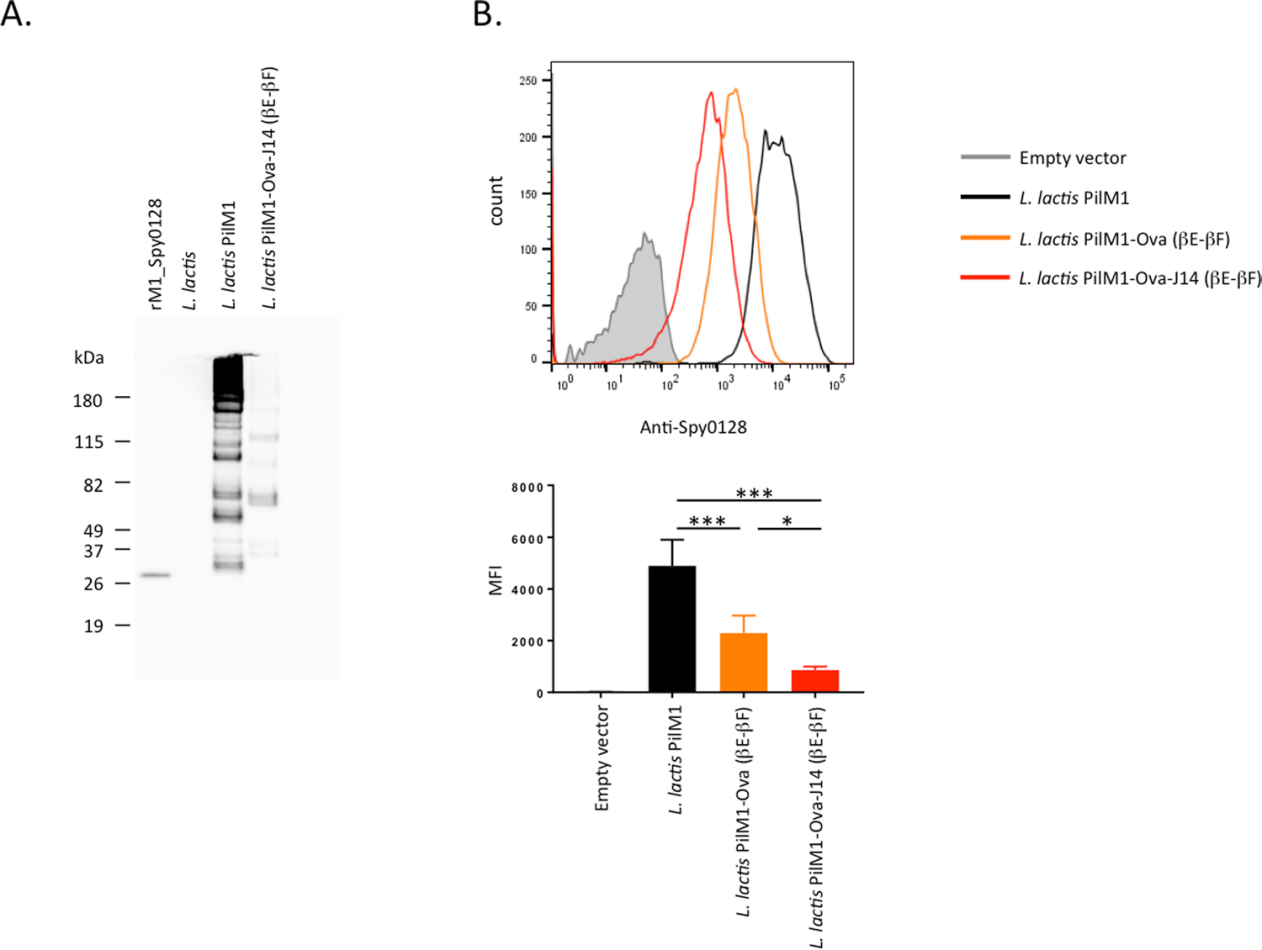
More than one peptide can be inserted in frame within a Spy0128 loop region. Cloning of a XhoI-SalI fragment into a *XhoI* site generates one non-cleavable hybrid site and one intact XhoI site downstream of the inserted DNA region. This allows the addition of consecutive DNA fragments (peptide sequences). A second peptide (J14) was added to the Ova_324–339_ peptide within the PilM1 βE-βF loop region (PilM1-Ova-J14). (**A**) Western blot analysis of *L*. *lactis* cell wall extracts (CWE) with antiserum specific for M1_Spy0128. The high molecular band patterns are indicative of pilus assembly. (**B**) Flow cytometry analysis shows expression of M1_Spy0128 on the surface of *L*. *lactis*, but with notably reduced expression after insertion of peptides into the βE-βF loop region. Error bars show the standard deviation from 3 independent experiments. *p<0.05; ***p<0.0005; one-way ANOVA followed by a Holm-Sidak test.

Our cloning strategy (peptides with flanking XhoI and SalI sites into the XhoI site of the *spy0128* gene) allows the insertion of multiple peptides in succession. XhoI and SalI digestions generate compatible restriction site overhangs, which result in a hybrid site after ligation that is not recognised by either XhoI or SalI. Therefore, cloning of the first peptide into the pilin gene neutralises one XhoI site and leaves the remaining XhoI site for cloning of a second peptide in frame with the first peptide. Using this strategy, we have inserted a second model peptide (J14, derived from the *S*. *pyogenes* M protein^31^), downstream of the Ova_324–339_ peptide in the βE-βF loop (Table 1a). This peptide fusion consists of a total of 47 amino acids and was successfully expressed within the PilM1 pilus on the surface of *L*. *lactis* as shown by the formation of high-molecular weight bands on Western blots (Fig. 5A). However, flow cytometry revealed that the expression level of Spy0128 was even lower (about 3 times) compared to the *L*. *lactis* PilM1-Ova construct with a single peptide in the βE-βF loop (Fig. 5B).

### Expression of peptides in the backbone pilins of other *S*. *pyogenes* pilus types

**Figure 6:**
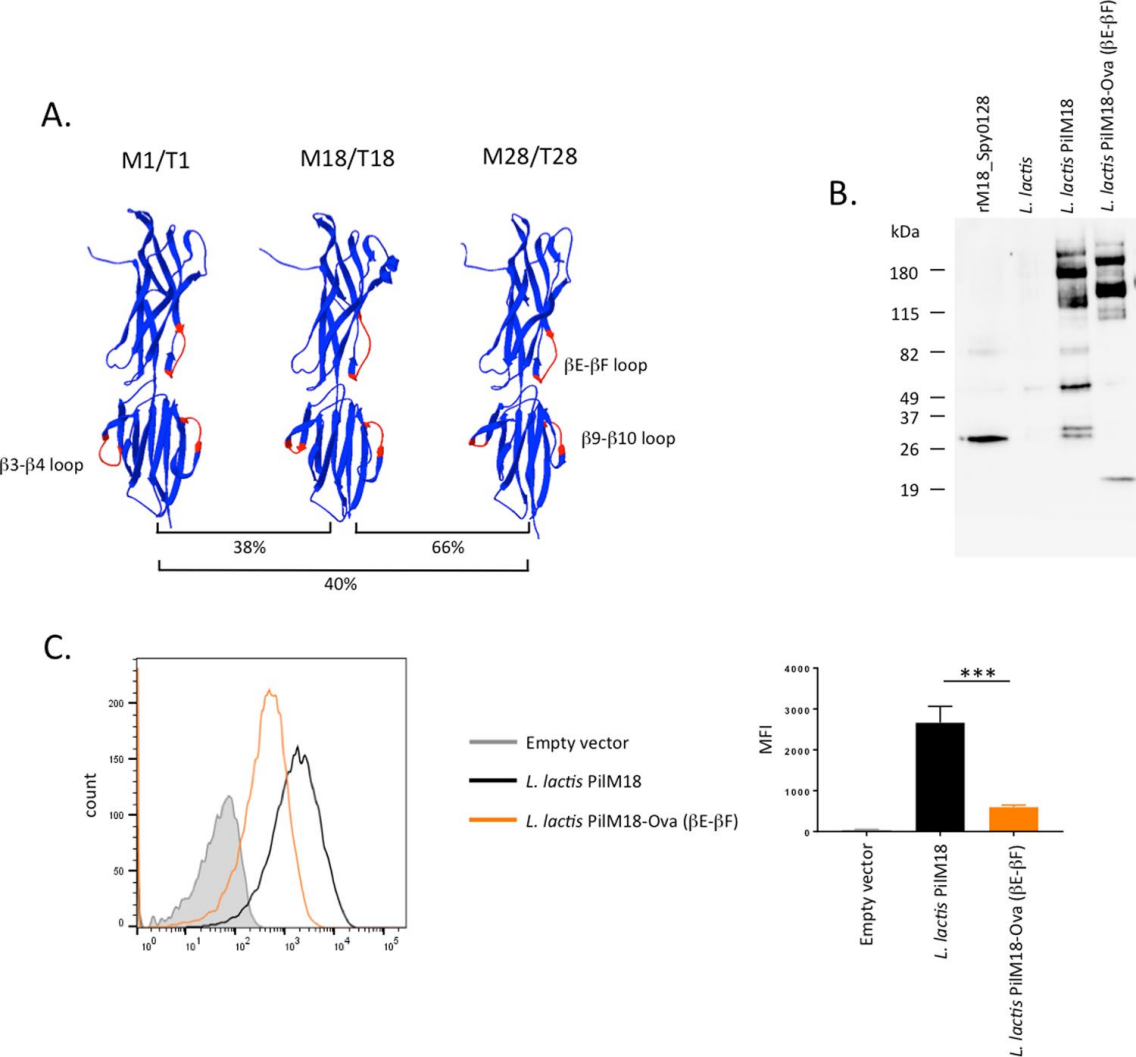
Peptides can be inserted into loop regions from structurally related backbone pilus proteins. (**A**) The structures of the backbone pilus proteins found in serotype M18/T18 and serotype M28/T28 were modelled onto the Spy0128 (serotype M1/T1) crystal structure (3B2M) using the Swiss PDB modeling server^53^. Despite low amino acid sequence identities, the models predicted conserved structures, which allowed the identification of loop regions equivalent to those analysed in Spy0128 (M1/T1). The model peptide Ova_324–339_ was inserted into the βE-βF loop region of M18_Spy0128 using the same cloning strategy as for M1_Spy0128. (**B**) Western blot analysis of *L*. *lactis* cell wall extracts (CWE) with antiserum specific for M18_Spy0128. (**C**) Flow cytometry analysis with anti-M18_Spy0128 antiserum shows expression of M18_Spy0128 on the surface of *L*. *lactis*, but with notably reduced expression after insertion of peptides into the βE-βF loop region. Error bars show the standard deviation from 3 independent experiments. ***p<0.0005; one-way ANOVA followed by a Holm-Sidak test.

Several antigenically different pilus types have been found in *S*. *pyogenes*^17^. The FCT-3 and FCT-4 pili each possess a backbone pilin with a predicted two-domain structure similar to the FCT-2 backbone pilin (Spy0128), despite rather low amino acid identities (38% and 40%, respectively). We used the SWISS PDB modeller to generate protein structure models of both the FCT-3 type (from *S*. *pyogenes* serotype M18) and the FCT-4 type (from *S*. *pyogenes* serotype M28) backbone pilins (Fig. 6A). This enabled us to identify the loop regions equivalent to the βE-βF, β3-β4 and β9-β10 loops in the M1_Spy0128. Insertion of the Ova_324–339_ peptide into the βE-βF loop region of M18_Spy0128 (Table 1b) resulted in expression and assembly of the M18 pilus (PilM18) on the surface of *L*. *lactis* (Fig. 6B). Similar to Spy0128 βE-βF-Ova, the insertion of the Ova_324–339_ peptide into the βE-βF loop region of M18_Spy0128 resulted in decreased expression of the backbone pilin (Fig. 6C).

## Discussion

Mucosal routes for vaccine delivery have gained an increased interest in modern vaccinology. Mucosal surfaces represent a major entry site of many pathogens. Hence, the ability of a vaccine to induce a local mucosal immune response is an efficient prophylactic strategy. Mucosal vaccination also has an advantage over parenterally administered vaccines in that it can elicit both mucosal and systemic immune responses, generally with reduced secondary effects^32^. Additionally, mucosal vaccines can be administered without needles and syringes, eliminating the need for trained personnel^33^.

There is growing emphasis on the use of peptide vaccines due to their specificity, thereby reducing unnecessary antigenic load and potentially allergenic and/or reactogenic responses^4^. However, the foremost challenge is that peptides are often poorly immunogenic on their own and undergo enzymatic degradation more quickly than folded proteins^34^. These features necessitate chemical coupling to a stabilising carrier, and/or administration with potent immunostimulatory adjuvants^35^, posing greater costs and potential safety concerns. To overcome these problems, we have developed a novel peptide carrier platform for the presentation of peptides as an integral part of the GAS serotype M1 pilus on the surface of *L*. *lactis*. *L*. *lactis* is a non-pathogenic bacterium which has a ‘generally recognised as safe’ (GRAS) status^36^ and has been thoroughly investigated as a delivery vehicle and adjuvant for mucosal vaccines^18,22,37,38^. However, *L*. *lactis* produces a cell envelope structural component known as a polysaccharide pellicle, similar to a bacterial capsule, which might restrict accessibility of the heterologous protein^39^. It has previously been proposed that foreign proteins expressed on the tip of a pilus might overcome this problem by exposing the protein at a distance from the cell envelope^27^. The pilus structure of GAS has been shown to be highly immunogenic, and *L*. *lactis* possesses natural adjuvancy that induces IL-2 and IFN-γ cytokines^40^. The combination of these two elements provides the basis of promising efficacy for the PilVax platform as a vaccine strategy.

The GAS serotype M1 pilus consists of 3 structural proteins that are covalently connected by a pilus assembly sortase on the cell surface to form a long protruding peritrichous structure. The length of the pilus is determined by the number of backbone pilins (Spy0128) incorporated into the pilus fibre, which is estimated to consist of around 50–100 Spy0128 monomers^10,15,17^. Our strategy was to increase peptide stability and immunogenicity by inserting the model peptide Ova_324–339_ into the compact (trypsin-resistant) and highly immunogenic backbone pilin, resulting in multimerisation of the peptide when the pilus structure is assembled (Supplementary Figure 1). Of the 8 potential integration sites within Spy0128 we analysed, 3 loop regions were found to be suitable for peptide expression. These include the βE-βF loop region in the N-terminal domain and the β3-β4 loop and β9-β10 loop regions in the C-terminal domain. Insertion of the model peptide Ova_324–339_ into the βB-βC, β2-β3 loop regions or the βG-β1 interdomain regions resulted in expression of monomeric Spy0128 in the protoplast fraction indicating a lack of pilus assembly. Notably, the Spy0128 proteins were slightly larger than the rSpy0128, which lacks the N-terminal secretion sequence and the C-terminal sortase C assembly domain. This suggests that lack of proper protein maturation is responsible for the failed pilus assembly. In contrast, insertion of the Ova peptide into the βD-βE loop or at the free N-terminal region between Asn28 and Gly29 did not result in detectable Spy0128 in any of the cell fractions or the cell culture supernatant. This suggests that the proteins either failed to express or were rapidly degraded after expression. The exact reason for the failed expression of pili was not further analysed as this is irrelevant to this study. In the successful constructs, pilus expression was generally lower than in *L*. *lactis* expressing unmodified PilM1 without peptide. The reduced pilus expression level could be explained by shorter pili being assembled, or lower amounts of pili being produced on the surface. As only the overall amount of Ova_324–339_-carrying Spy0128 pilins is important for our study, not the length of the pili, we have not investigated this any further.

One of the successful constructs, *L*. *lactis* expressing PilM1 with Ova_324–339_ at the βE-βF loop region (*L*. *lactis* PilM1-Ova) was used to intranasally immunise groups of mice and to test for Ova-specific antibody responses. Our study shows that *L*. *lactis* PilM1-Ova induces detectable Ova-specific IgA responses at mucosal sites (measured in saliva and BAL fluid) and in serum after intranasal vaccination. Antibody responses were measurable against ovalbumin by ELISA, indicating that the antibodies recognised the Ova_324–339_ peptide as part of the complete protein. This suggests that PilVax might be a suitable peptide delivery platform to develop mucosal vaccines against respiratory pathogens. In addition, we found detectable Ova-specific IgG responses in serum samples of immunised mice. As systemic antibodies can reduce bacterial spreading from mucosal sites and prevent systemic disease^41^, this is an important finding demonstrating the suitability of developing the PilVax platform into effective vaccines. Furthermore, serum-derived IgG can also contribute significantly to immune defence in the lower respiratory tract and in the genitourinary mucosa^42^. Notably, Ova-specific IgG or IgA were not detected in serum or mucosal sites when synthetic Ova_324–339_ was mixed with *L*. *lactis* PilM1. This indicates that the adjuvant property of *L*. *lactis* alone is not sufficient to induce Ova-specific immunity and suggests that the physical integration of the peptide into the pilus structure is important, probably due to peptide stabilisation and prevention of enzymatic degradation. The Ova-specific IgA response in BAL fluid was similar between *L*. *lactis* PilM1-Ova and Ova + CT-B immunised mice, even though the amount of synthetic Ova peptide was approximately 36,000-times higher in the Ova + CT-B group. This might again be due to the increased stability of the peptide when incorporated into the rigid pilus structure. Interestingly, no significant Ova-specific IgA titers were found in the serum after immunisation with Ova + CT-B. However, the reason for this is unclear.

Further investigation into the type of immune response generated by *L*. *lactis* PilM1-Ova showed a mixed IgG1/IgG2a systemic response. This was in contrast to the control immunisation with synthetic Ova_324–339_ mixed with CT-B adjuvant, which generated a predominant IgG1 response. In mice, IgG1 is associated with a Th2-like response, while a Th1 response promotes the induction of IgG2a antibodies^43^. Yanase *et al*. recently reported that ovalbumin conjugated to nanoparticles (Ova-NPs) also stimulated a mixed IgG1/IgG2a Ova-specific response, and only low levels of unwanted and potentially toxic IgE antibodies in mice. In contrast, immunisation with Ova in alum stimulated Ova-specific IgG1 and IgE, but only low levels of IgG2a/IgG2b antibodies^44^. Notably, IgA was not detected in either of these schedules, likely due to the subcutaneous route of immunisation used in this study^44^.

We have further shown that it is possible to insert more than one peptide into the same integration site, expanding the versatility of the PilVax platform. Introducing unique restriction enzyme recognition sites into each of the 3 accommodating loop regions within the same construct could also further increase the number of different peptides that could be presented. Finally, we have shown that peptide epitopes can be incorporated into structurally similar, but antigenically different pilus structures, However, PilM18 expression was much lower than observed with PilM1 and therefore the suitability of this pilus type for peptide-presentation requires further evaluation.

One drawback of the PilVax platform is that peptide insertion, at least for the model Ova_324–339_ peptide tested in this study, resulted in notable reduction in pilus expression level on the *L*. *lactis* surface. However, we have shown that significant Ova-specific IgA and IgG levels could be generated in mice immunised with the construct that had the lowest pilus expression (*L*. *lactis* PilM1-Ova βE-βF). Based on the assumption that higher pilus/peptide expression would lead to improved immune responses it would be advisable that for any future application with a vaccine peptide, all integration sites should be investigated in order to identify the construct that shows the highest pilus expression levels.

Although live *L*. *lactis* vectors have been investigated as vaccines against a variety of different antigens from various pathogens^18–22,37,38,45,46^, the lack of a biological containment strategy has hindered progress into clinical trials. One solution is the use of an auxotroph mutant to prevent selection by a plasmid-encoded antibiotic resistance gene, e.g. the *L*. *lactis* NZ9130 strain, which carries an alanine racemase gene deletion preventing growth in the absence of alanine^47,48^. More recently, a non-GMO alternative to live *L*. *lactis* has been introduced which is based on hot acid-treated nucleotide-free *L*. *lactis* known as bacterium-like particles (BLP)^49,50^.

In conclusion, we have developed a novel peptide delivery platform that might be useful for mucosal vaccinations against a variety of pathogens that colonise mucosal sites, such as *Streptococcus pneumoniae*, *Neisseria gonorrhoeae*, *Chlamydia trachomatis* and *Staphylococcus aureus*. As proof of concept, we have shown that insertion of the model peptide Ova_324–339_ into the monomeric pilus backbone protein Spy0128 increases immunogenicity most likely due to peptide stabilisation and multimerisation. Other benefits of PilVax include low production costs, as no chemical coupling is required and the modified bacteria can easily be shipped in lyophilised form. Furthermore, no potentially toxic adjuvants are required and the vaccine can be delivered needle-free to a mucosal site. These advantages in safety, low production cost and ease of administration are highly desired in developing countries, where efficacious vaccines are most needed.

## Methods

### Bacterial strains and culture conditions

*S*. *pyogenes* strains SF370 (serotype M1) and MGAS8232 (serotype M18) were grown stationary in Brain Heart Infusion (BHI) broth (BD) at 37 °C, *L*. *lactis* MG1363 was grown stationary in M17 media (BD) supplemented with 0.5% glucose at 28 °C, and *E*. *coli* DH5α was grown aerobically at 37 °C in LB broth (HiMedia). When required, antibiotics were added: ampicillin (50 μg/ml, *E*. *coli*), kanamycin (50 μg/ml, *E*. *coli*; 200 μg/ml, *L*. *lactis*).

### Genetic manipulations

The complete pilus operon was amplified from *S*. *pyogenes* SF370 or MGAS8232 genomic DNA by PCR using iProof DNA polymerase (Roche) and primers shown in Table 2. The purified PCR products were digested with BamHI (PilM1) or BamHI/PstI (PilM18) and cloned into plasmid pLZ12km2_P23R^51,52^ to generate pLZ12km2_P23R:PilM1 and pLZ12km2_P23R:PilM18.

**Table 2:** List of oligos used in this study

Name	Sequence (5′ to 3′)
PilM1.fw	GC**GGATCC**GATATGATGTCACATTGAGAG
PilM1.rev	GCG**GATCC**GTCGTGGGGCAATAAAAAATTC
PilM18.fw	GC**GGATCC**GACCATATACACGGTTGCAGGA
PilM18.rev	CGG**CTGCAG**TCTTATATGGTACAAAAACCTATCAGC
PilM28.fw	GC**GGATCC**GATTTTATTCAGAAGGAGAGG
PilM28.rev	CGG**CTGCAG**TCTTATATGGTACAAAAACCTATCAGC
PilM1_N29.fw	CCCG**CTCGAG**GGAGCCAAACTAACAGTTAC
PilM1_N29.rev	CCCG**CTCGAG**GTTTACAACAGTCTCCCC
PilM1_βG-β1.fw	GAGAGA**CTCGAG**ACATTAACGGTGAAGAAAAAAG
PilM1_βG-β1.rev	GAGAGA**CTCGAG**ATCTAAGCTATTTTTGAACTG
PilM1_βB-βC.fw	GAGAGAGA**CTCGAG**GGAAATAAGTTTAAAGGTGTAGC
PilM1_βB-βC.rev	GAGAGAGA**CTCGAG**AGTATCAGGTTCGATTTTAAATG
PilM1_βD-βE.fw	GAGAGAGA**CTCGAG**GGTGTTTATTATTACAAAG
PilM1_βD-βE.rev	GAGAGAGA**CTCGAG**AAAATCAAATTCTGCAG
PilM1_βE-βF.fw	GAGAGAGA**CTCGAG**GGTGTTTCTTATGATACAAC
PilM1_βE-βF.rev	GAGAGAGA**CTCGAG**CTCCTCAGTTACTTTG
PilM1_β2-β3.fw	GAGAGAGA**CTCGAG**TCAGAAAAAGTCATGATTGAG
PilM1_β2-β3.rev	GAGAGAGA**CTCGAG**TGCTTTTAAAGTCAGACC
PilM1_β3-β4.fw	GAGAGAGA**CTCGAG**CCTGTTCAAACAGAGGCTAG
PilM1_β3-β4.rev	GAGAGAGA**CTCGAG**AGTTGTCTTCTCAATCATGAC
PilM1_β9-β10.fw	GAGAGAGA**CTCGAG**AAAAATATCGCAGGTAATTC
PilM1_β9-β10.rev	GAGAGAGA**CTCGAG**TTGAGGACTAACTTCCACG
PilM18_βE-βF.fw	GAGAGAGA**CTCGAG**ATAGCTTATGATTCTCAAC
PilM18_βE-βF.fw	GAGAGAGA**CTCGAG**TACTTCAGAAACCGTATAAC
PilM28_βE-βF.fw	GAGAGAGA**CTCGAG**GGGATTAAATACGATACC
PilM28_βE-βF.rev	GAGAGAGA**CTCGAG**TATTTCTGAAACCGTATAAC
Ova_324–339_.fw	CCCG**CTCGAG**TCACAAGCTGTTCATGCTGCACATGCAGAAAT
Ova_324–339_.rev	CCCG**GTCGAC**TCTACCTGCTTCATTAATTTCTGCATGTGCAGC
J14.fw	CCCG**CTCGAG**AAACAAGCTGAAGATAAAGTTAAAGCTTCACGTGAAGCCAAAAAGCAAGTCG
J14.rev	CCCG**GTCGAC**TTGAACTTTATCTTCAAGTTGTTCAAGAGCTTTTTCGACTTGCTTTTTGGC
PilM1_linker.fw	ACGC**GTCGAC**GGCAGCGGTAGCGGC CTCGAG GGC
PilM1_linker.rev	ACGC**GTCGAC**GCCGCTACCGCTGCC CTCGAG GCCG

Primer sequences used in this study. Restriction sites are shown in bold letters.

The unique XhoI in the vector’s multiple cloning site was first deleted, then re-introduced by full circle PCR with specific primers shown in Table 2 followed byXhoI digestion and religation. This allowed the insertion of peptide-encoding DNA sequences with flanking XhoI or SalI restriction sites. The DNA encoding Ova_324–339_ and J14^31^ peptides were generated by PCR with overlapping primers (see Table 2), digested with XhoI and SalI and cloned into selected XhoI sites previously introduced into the pilus backbone genes as described above.

DNA sequences were confirmed by Sanger sequencing using the DNA sequencing service at the School of Biological Sciences (The University of Auckland, New Zealand). *L*. *lactis* strain MG1363 was transformed by electroporation with the resulting plasmids at 2100 V, 25 μF, 200 Ω in a 2 mm cuvette using a Gene Pulser Xcell^TM^ (Bio-Rad).

### Bioinformatic analysis

The structural model of the FCT-3 backbone pilin was generated using the Swiss PDB modelling server at the Swiss Institute of Bioinformatics^53^ with Spy0128 (3B2M) as a template. The structural images were created with the PDB Swiss Viewer version 4.1.0.^54^.

### Cell wall extracts and Western blots

An overnight *L*. *lactis* culture was spun down at 4,500 rpm for 15 min at 4 °C. The bacterial cell pellet was washed once with PBS, then resuspended at 0.1 g/ml in cold protoplast buffer (40% sucrose, 10 mM MgCl_2_, 0.1 M KPO_4_, 2 mg/ml lysozyme, 400 U mutanolysin, Roche EDTA-free protease inhibitor) and incubated for 3 h at 37 °C with constant rotation. Cellular debris was removed by centrifugation at 13,000 × *g* for 15 min at 4 °C, and the supernatant containing cell surface proteins were run on a NuPAGE^TM^ Novex^TM^ 4–12% gradient gel (Invitrogen). Separated proteins were transferred onto a nitrocellulose (N+) membrane (Biotrace NT, Pall Life Science, USA) in NuPAGE™ Transfer Buffer (Invitrogen) using a Hoefer TE77 Semi-phor semi-dry transfer unit. The membrane was blocked with 5% (w/v) non-fat milk in TBS, 0.5% (v/v) tween 20 (TBS-T), then probed with polyclonal rabbit antibodies against M1_Spy0128, M18_Spy0128^26^ or serum from Ova + CT-B immunised mice. After intensive washing in TBS-T, the membrane was then probed with goat anti-rabbit or goat anti-mouse HRP-conjugated antibody. The membrane was washed again before being developed using Amersham ECL Prime Western blotting detection reagent (GE Healthcare). Chemiluminescence signals were captured and digitised into image using a ChemiDoc™ Imaging System (Bio-Rad).

### Flow cytometry

*L*. *lactis* strains were grown overnight in GM17 medium, harvested by centrifugation (5000 × *g*, 5 min), and resuspended in 1 ml blocking buffer (PBS/3% FBS/5 mM EDTA) at an OD_600_ of 0.4. Cells were dispersed in a water bath sonicator for 2 min, then incubated on ice for 30 min. Blocking buffer was removed by centrifugation, and bacteria were washed once with FACS buffer (PBS/1% FBS/5 mM EDTA) and incubated with anti-Spy0128 antibody in FACS buffer on ice for 30 min. Bacteria were washed once, then incubated with anti-rabbit IgG FITC (Abacus ALS Ltd.) on ice for 30 min. Flow cytometry analysis was performed on a LSRII Flow Cytometer and analysed using FlowJo software.

### Immunisations

Frozen aliquots of *L*. *lactis* were prepared as previously described^26^ and enumerated by plating. On immunisation days, an aliquot was thawed at RT, washed and resuspended in PBS. Five to six week old Balb/c mice (n = 5) were immunised intranasally, under isoflurane anaesthesia, with 1 × 10^8^ CFU live recombinant *L*. *lactis* with or without 1.4 ng Ova peptide, 1.4 ng Ova peptide alone, or 50 μg Ova peptide + 4 μg CT-B, in a 10 μl volume. The bacterial suspensions were administered on 3 consecutive days (day 0, 1 and 2) as described previously^27^. An additional 3 boosters were given, 1–2 weeks apart, for a total of 12 doses (days 0, 1, 2, 14, 15, 16, 28, 29, 30, 35, 36, 37). Pre-immune serum was collected on day 0 and immune serum was collected 1 week after final immunisation (day 44). Bronchoalveolar lavage (BAL) was performed with 1 ml PBS, and saliva samples collected by rinsing the oral cavity with 50 μl PBS on day 44.

### ELISA

MaxiSorp plates (Nunc) were coated with 1 μg/ml recombinant Spy0128 protein or commercial ovalbumin protein (Invivogen) in PBS overnight at 4 °C, prior to incubation with titrated serum, BAL fluid or saliva. Detection was performed using goat anti-mouse IgG/IgG1/IgG2a-HRP (Life Technology) or goat anti-mouse IgA-HRP (Invitrogen) and 3,3,5,5-tetramethylbenzidine (ThermoFisher). Absorbance was determined on an EnSpire multilabel plate reader (Perkin Elmer). Endpoint titres were determined as the minimum serum dilution above the control (absorbance of 1:200 dilution of pre-immune serum plus 3 times the standard deviation).

### Animal Ethics

All animal experiments were performed in the Vernon Jansen Unit (University of Auckland, New Zealand) in accordance with relevant guidelines and regulations approved by the University of Auckland Animal Ethics Committee.

### Data availability

The datasets generated during and/or analysed during the current study are available from the corresponding author on reasonable request.

## Electronic supplementary material

Supplemental Materials

## References

[1] Ehreth J (2003). The global value of vaccination. Vaccine.

[2] Clark TG, Cassidy-Hanley D (2005). Recombinant subunit vaccines: potentials and constraints. Develop Biol.

[3] Hansson M, Nygren PA, Stahl S (2000). Design and production of recombinant subunit vaccines. Biotech Appl Biochem.

[4] Li W, Joshi MD, Singhania S, Ramsey KH, Murthy AK (2014). Peptide. Vaccine: Progress and Challenges. Vaccines.

[5] Lee VH (1988). Enzymatic barriers to peptide and protein absorption. Crit Rev Ther Drug Carrier Sys.

[6] Purcell AW, McCluskey J, Rossjohn J (2007). More than one reason to rethink the use of peptides in vaccine design. Nat Rev Drug Discov.

[7] Bijker MS, Melief CJ, Offringa R, van der Burg SH (2007). Design and development of synthetic peptide vaccines: past, present and future. Exp Rev Vaccines.

[8] Vartak, A. & Sucheck, S. J. Recent Advances in Subunit Vaccine Carriers. *Vaccines* **4**, 10.3390/vaccines4020012 (2016). 10.3390/vaccines4020012 PMC4931629 27104575

[9] Danne C, Dramsi S (2012). Pili of gram-positive bacteria: roles in host colonization. Res Microbiol.

[10] Proft T, Baker EN (2009). Pili in Gram-negative and Gram-positive bacteria - structure, assembly and their role in disease. Cell Mol Life Sci.

[11] Hendrickx APA, Budzik JM, Oh S-Y, Schneewind O (2011). Architects at the bacterial surface — sortases and the assembly of pili with isopeptide bonds. Nat Rev Microbiol.

[12] Bessen DE, Kalia A (2002). Genomic localization of a T serotype locus to a recombinatorial zone encoding extracellular matrix-binding proteins in *Streptococcus pyogenes*. Infect Immun.

[13] Kreikemeyer B (2005). *Streptococcus pyogenes* collagen type I-binding Cpa surface protein. Expression profile, binding characteristics, biological functions, and potential clinical impact. J Biol Chem.

[14] Linke-Winnebeck C (2014). Structural model for covalent adhesion of the *Streptococcus pyogenes* pilus through a thioester bond. J Biol Chem.

[15] Kang HJ, Coulibaly F, Clow F, Proft T, Baker EN (2007). Stabilizing isopeptide bonds revealed in Gram-positive bacterial pilus structure. Science.

[16] Linke C (2010). Crystal structure of the minor pilin FctB reveals determinants of Group A streptococcal pilus anchoring. J Biol Chem.

[17] Mora M (2005). Group A Streptococcus produce pilus-like structures containing protective antigens and Lancefield T antigens. Proc Natl Acad Sci USA.

[18] Bahey-El-Din M (2012). *Lactococcus lactis*-based vaccines from laboratory bench to human use: an overview. Vaccine.

[19] Bahey-El-Din M, Gahan CG, Griffin BT (2010). *Lactococcus lactis* as a cell factory for delivery of therapeutic proteins. Curr Gene Ther.

[20] Berlec A, Ravnikar M, Strukelj B (2012). Lactic acid bacteria as oral delivery systems for biomolecules. Die Pharmazie.

[21] Bermudez-Humaran LG, Kharrat P, Chatel JM, Langella P (2011). Lactococci and lactobacilli as mucosal delivery vectors for therapeutic proteins and DNA vaccines. Microb Cell Fact.

[22] Mercenier A, Muller-Alouf H, Grangette C (2000). Lactic acid bacteria as live vaccines. Curr Iss Mol Biol.

[23] Braat H (2006). A phase I trial with transgenic bacteria expressing interleukin-10 in Crohn’s disease. Clin Gastroenterol Hepatol.

[24] Becherelli M (2012). The ancillary protein 1 of *Streptococcus pyogenes* FCT-1 pili mediates cell adhesion and biofilm formation through heterophilic as well as homophilic interactions. Mol Microbiol.

[25] Edwards AM (2008). Scavenger receptor gp340 aggregates group A streptococci by binding pili. Mol Microbiol.

[26] Loh, J. M. S., Lorenz, N., Tsai, C. J.-Y., Khemlani, A. & Proft, T. Mucosal vaccination with pili from Group A Streptococcus expressed on *Lactococcus lactis* generates protective immune responses. *Sci Rep* (2017). 10.1038/s41598-017-07602-0 PMC5543120 28775292

[27] Quigley BR (2010). A foreign protein incorporated on the Tip of T3 pili in Lactococcus lactis elicits systemic and mucosal immunity. Infect Immun.

[28] Young PG, Proft T, Harris PW, Brimble MA, Baker EN (2014). Structure and activity of *Streptococcus pyogenes* SipA: a signal peptidase-like protein essential for pilus polymerisation. PloS one.

[29] Johnsen G, Elsayed S (1990). Antigenic and allergenic determinants of ovalbumin–III. MHC Ia-binding peptide (OA 323–339) interacts with human and rabbit specific antibodies. Mol Immunol.

[30] McFarland BJ, Sant AJ, Lybrand TP, Beeson C (1999). Ovalbumin(323–339) peptide binds to the major histocompatibility complex class II I-A(d) protein using two functionally distinct registers. Biochemistry.

[31] Olive C, Clair T, Yarwood P, Good MF (2002). Protection of mice from group A streptococcal infection by intranasal immunisation with a peptide vaccine that contains a conserved M protein B cell epitope and lacks a T cell autoepitope. Vaccine.

[32] Bermudez-Humaran LG (2005). A novel mucosal vaccine based on live Lactococci expressing E7 antigen and IL-12 induces systemic and mucosal immune responses and protects mice against human papillomavirus type 16-induced tumors. J Immunol.

[33] Yuki Y, Kiyono H (2009). Mucosal vaccines: novel advances in technology and delivery. Expert Rev Vaccines.

[34] Kaumaya PT (1993). Peptide vaccines incorporating a ‘promiscuous’ T-cell epitope bypass certain haplotype restricted immune responses and provide broad spectrum immunogenicity. J Mol Recog: JMR.

[35] O’Hagan DT, De Gregorio E (2009). The path to a successful vaccine adjuvant–‘the long and winding road’. Drug Discov Today.

[36] Song AA, In LL, Lim SH, Rahim RA (2017). A review on *Lactococcus lactis*: from food to factory. Microb Cell Fact.

[37] Thole JE (2000). Live bacterial delivery systems for development of mucosal vaccines. Curr Opin Mol Therap.

[38] Wyszynska A, Kobierecka P, Bardowski J, Jagusztyn-Krynicka EK (2015). Lactic acid bacteria–20 years exploring their potential as live vectors for mucosal vaccination. Appl Microbiol Biotechnol.

[39] Chapot-Chartier MP (2010). Cell surface of *Lactococcus lactis* is covered by a protective polysaccharide pellicle. J Biol Chem.

[40] Bermudez-Humaran LG (2004). An inducible surface presentation system improves cellular immunity against human papillomavirus type 16 E7 antigen in mice after nasal administration with recombinant lactococci. J Med Microbiol.

[41] Lima FA (2012). Controlled inflammatory responses in the lungs are associated with protection elicited by a pneumococcal surface protein A-based vaccine against a lethal respiratory challenge with *Streptococcus pneumoniae* in mice. Clin Vaccine Immunol: CVI.

[42] Holmgren J, Czerkinsky C (2005). Mucosal immunity and vaccines. Nat Med.

[43] Germann T (1995). Interleukin-12 profoundly up-regulates the synthesis of antigen-specific complement-fixing IgG2a, IgG2b and IgG3 antibody subclasses *in vivo*. Eur J Immunol.

[44] Yanase N (2014). OVA-bound nanoparticles induce OVA-specific IgG1, IgG2a, and IgG2b responses with low IgE synthesis. Vaccine.

[45] Chamcha V, Jones A, Quigley BR, Scott JR, Amara RR (2015). Oral Immunization with a Recombinant *Lactococcus lacti*s-Expressing HIV-1 Antigen on Group A Streptococcus Pilus Induces Strong Mucosal Immunity in the Gut. J Immunol (Baltimore, Md.: 1950).

[46] Trombert A (2015). Recombinant lactic acid bacteria as delivery vectors of heterologous antigens: the future of vaccination?. Beneficial Microbes.

[47] Bron PA (2002). Use of the alr gene as a food-grade selection marker in lactic acid bacteria. Appl Environ Microbiol.

[48] Lu W, Kong J, Kong W (2013). Construction and application of a food-grade expression system for *Lactococcus lactis*. Mol Biotech.

[49] Lu J (2017). Systemic and mucosal immune responses elicited by intranasal immunization with a pneumococcal bacterium-like particle-based vaccine displaying pneumolysin mutant Plym2. Immunol Lett.

[50] van Roosmalen ML (2006). Mucosal vaccine delivery of antigens tightly bound to an adjuvant particle made from food-grade bacteria. Methods (San Diego, Calif.).

[51] Loh JM, Proft T (2013). Toxin-antitoxin-stabilized reporter plasmids for biophotonic imaging of Group A streptococcus. Appl Microbiol Biotech.

[52] Okada N, Tatsuno I, Hanski E, Caparon M, Sasakawa C (1998). *Streptococcus pyogenes* protein F promotes invasion of HeLa cells. Microbiology (Reading, England).

[53] Arnold K, Bordoli L, Kopp J, Schwede T (2006). The SWISS-MODEL workspace: a web-based environment for protein structure homology modelling. Bioinformatics (Oxford, England).

[54] Johansson MU, Zoete V, Michielin O, Guex N (2012). Defining and searching for structural motifs using DeepView/Swiss-PdbViewer. BMC Bioinformatics.

