# Supplementary material for: PilVax – a novel peptide delivery platform for the development of mucosal vaccines
Source: Sci Rep. 2018 Feb 7;8:2555. doi: 10.1038/s41598-018-20863-7 (PMC5803258; doi:10.1038/s41598-018-20863-7)
Supplement: Supplementary file 1 — Supplemental Materials [file 41598_2018_20863_MOESM1_ESM.pdf]

Supplementary Data

PilVax – a novel peptide delivery platform for the development of mucosal vaccines

Dasun Wagachchi[1,2], Jia-Yun C. Tsai[1,2], Callum Chalmers[1], Sam Blanchett[1], Jacelyn M. S. Loh[1,2*], Thomas Proft[1,2*]

[1]Department of Molecular Medicine & Pathology, School of Medical Sciences, University of Auckland, Auckland 1023, New Zealand.

[2]Maurice Wilkins Centre for Molecular Biodiscovery, Auckland 1023, New Zealand.

*corresponding author email: TP (t.proft@auckland.ac.nz) or JMSL (email: mj.loh@auckland.ac.nz).

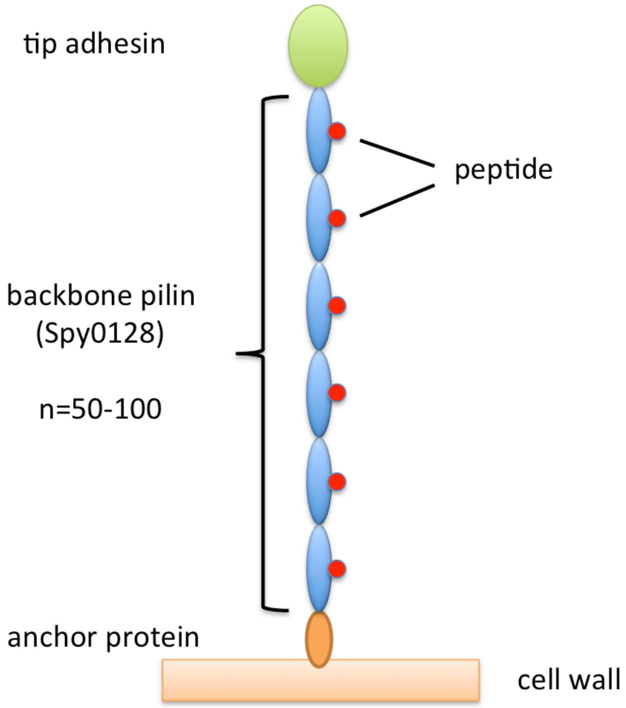

Supplementary Figure 1. Schematic diagram of the GAS M1 pilus with inserted peptide. The peritrichous GAS M1 pili are formed from 3 structural proteins (pilins) covalently linked by a pilus-specific transpeptidase (sortase C). Approximately 50-100 copies of the backbone pilin (Spy0128) mutlimerise to form the elongated pilus fibre, which is decorated by a tip adhesin and linked to the bacterial cell wall by an anchor protein. Genetic engineering of a peptide-encoding DNA sequence into the *spy0128* gene results in expression of a large number of well-exposed peptides (red circle) on the pilus fibre. Spy0128 has a compact protease-resistant protein structure, which stabilises the inserted peptide to prevent enzymatic degradation.

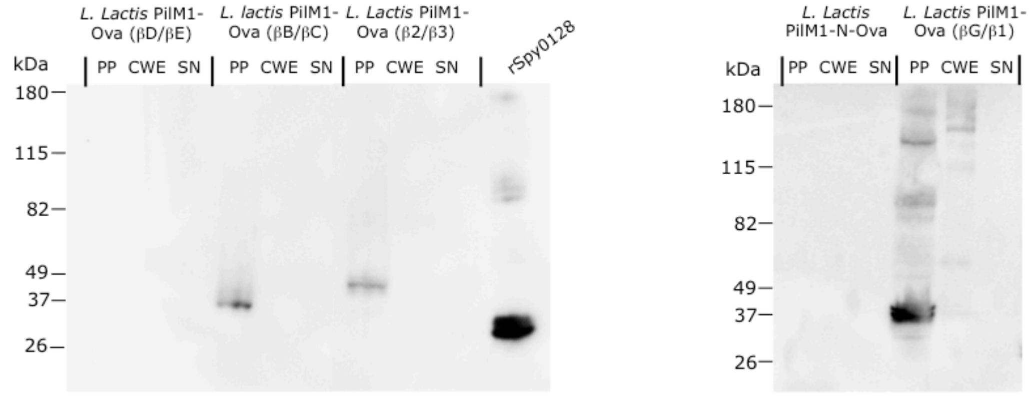

Supplementary Figure 2. Westen blot analysis of *L. lactis* PilM1-ova cell fractions.
An overnight *L. lactis* culture was spun down at 4,500 rpm for 15 min at 4°C. The bacterial cell pellet was washed once in PBS, then resuspended at 0.1 g/ml in cold protoplast buffer (40% sucrose, 10 mM $MgCl_2$, 0.1 M KPO_4, 2 mg/ml lysozyme, 400 U mutanolysin, Roche EDTA-free protease inhibitor) and incubated for 3 h at 37°C with constant rotation. The protoplast (PP) fraction was separated from the cell wall extract (CWE) by centrifugation at 13,000 xg for 15 min at 4°C. The cell fractions (PP and CWE) and cell culture supernatant (SN) were run on a NuPAGE^{TM} Novex^{TM} 4-12% gradient gel (Invitrogen). Separated proteins were transferred onto a nitrocellulose (N+) membrane (Biotrace NT, Pall Life Science, USA) in NuPAGE™ Transfer Buffer (Invitrogen) using a Hoefer TE77 Semi-phor semi-dry transfer unit. The membrane was blocked with 5% (w/v) non-fat milk in TBS, 0.5% (v/v) tween 20 (TBS-T), then probed with polyclonal rabbit antibodies against M1_Spy0128. After intensive washing in TBS-T, the membrane was then probed with goat anti-rabbit HRP-conjugated antibody. The membrane was washed again before being developed using Amersham ECL Prime Western blotting detection reagent (GE Healthcare). Chemiluminescence signals were captured and digitised into image using a ChemiDoc™ Imaging System (Bio-Rad).
